# Only Behavioral But Not Self-Report Measures of Speech Perception Correlate with Cognitive Abilities

**DOI:** 10.3389/fpsyg.2016.00576

**Published:** 2016-05-23

**Authors:** Antje Heinrich, Helen Henshaw, Melanie A. Ferguson

**Affiliations:** ^1^Medical Research Council Institute of Hearing Research, NottinghamUK; ^2^Otology and Hearing Group, National Institute for Health Research Nottingham Hearing Biomedical Research Unit, Division of Clinical Neuroscience, School of Medicine, University of Nottingham, NottinghamUK; ^3^Nottingham University Hospitals NHS Trust, NottinghamUK

**Keywords:** speech perception, cognition, self-report, communication, hearing aid users, mild-to-moderate hearing loss

## Abstract

Good speech perception and communication skills in everyday life are crucial for participation and well-being, and are therefore an overarching aim of auditory rehabilitation. Both behavioral and self-report measures can be used to assess these skills. However, correlations between behavioral and self-report speech perception measures are often low. One possible explanation is that there is a mismatch between the specific situations used in the assessment of these skills in each method, and a more careful matching across situations might improve consistency of results. The role that cognition plays in specific speech situations may also be important for understanding communication, as speech perception tests vary in their cognitive demands. In this study, the role of executive function, working memory (WM) and attention in behavioral and self-report measures of speech perception was investigated. Thirty existing hearing aid users with mild-to-moderate hearing loss aged between 50 and 74 years completed a behavioral test battery with speech perception tests ranging from phoneme discrimination in modulated noise (easy) to words in multi-talker babble (medium) and keyword perception in a carrier sentence against a distractor voice (difficult). In addition, a self-report measure of aided communication, residual disability from the Glasgow Hearing Aid Benefit Profile, was obtained. Correlations between speech perception tests and self-report measures were higher when specific speech situations across both were matched. Cognition correlated with behavioral speech perception test results but not with self-report. Only the most difficult speech perception test, keyword perception in a carrier sentence with a competing distractor voice, engaged executive functions in addition to WM. In conclusion, any relationship between behavioral and self-report speech perception is not mediated by a shared correlation with cognition.

## Introduction

Good communication skills in everyday life are crucial for wellbeing and are therefore overarching aims of audiological rehabilitation. Communication abilities can be measured in a variety of ways, and the measures do not necessarily assess identical or even overlapping aspects of communication. One way of measuring communication abilities is by using speech perception tests. They use behavioral indices to assess the passive perception of speech without an opportunity for interaction with other people. We use the term speech perception tests in accordance with Erber’s (1982) framework that defines perception as the identification and repeatability of phrases without deeper comprehension. Note that speech perception represents but one aspect of communication. In real-life situations communication includes additional aspects such as the bi-directional transfer of information (Kiessling et al., 2003). Better suited to assess this second aspect of communication are self-report questionnaires. In contrast to behavioral speech perception tests, they often explicitly ask about how acoustic and linguistic information is used and transmitted effectively in a bi-directional process. Given this difference, it is likely that these two measures assess only partially complementary aspects of a listener’s experience (see Pronk et al., 2011 for a similar argument).

### Behavioral versus Self-Report Measures to Assess Speech Perception and Communication

Correlations between behavioral and self-report measures of speech perception and communication vary substantially across studies from hardly any correlations in some studies to consistent correlations in other studies. Hardly any correlations were found by Newman et al. (1990) when testing middle-aged non-hearing aid users^1^ on the Hearing Handicap Inventory for Adults and an unspecified word recognition test. In contrast, consistently high correlations between a speech-perception-in-noise measure (SRTN) and all subscales of the Amsterdam Inventory for Auditory Disability and Handicap were found in a group of older hearing aid users and non-hearing aid users by Zekveld et al. (2013). In other studies, the correlation strength depended on the particular combination of self-report subscales and speech perception tests (Cox and Alexander, 1992; Ng et al., 2013; Heinrich et al., 2015). For instance, Cox and Alexander (1992) compared intelligibility in the Connected Speech Test for a number of simulated listening environments with the subscales of the Profile of Hearing Aid Benefit questionnaire, and found correlations only between two speech perception tasks and two questionnaire subscales. All other combinations of behavioral and self-report measures did not yield significant correlations. Similarly, inconsistent results were found by Ng et al. (2013), who tested a sample of hearing aid users with an SRTN test and the International Outcome Inventory for Hearing Aids (IOI-HA) and the Speech, Spatial and Qualities of Hearing (SSQ) Scale. While they found no significant correlations between speech perception and self-report measures when either the IOI-HA or SSQ subscales of complex speech perception (Speech in noise, Speech in speech contexts, Multiple speech-streams processing and switching) were used, they did find significant correlations with other aspects of self-reported listening (i.e., quality and spatial listening). Finally, inconsistent correlations were also found by Heinrich et al. (2015) when testing older non-hearing aid users. They tested intelligibility in a range of speech perception situations and with different methods of setting signal-to-noise ratios (SNRs) and compared these behavioral results to a variety of self-report measures. The only instance in which they found consistent correlations between almost all self-report questionnaires and a word perception test was when the SNR was changed by adjusting the background noise level, but not when the SNR was changed by adjusting the target speech. A consequence of the former adjustment method was an overall increase of the overall presentation level of the speech test, whereas the latter method led to a decrease in overall presentation level. This finding suggests that only speech perception test methods that altered the background noise, as opposed to altering the speech levels, capture aspects of communication, participation restriction and tolerance to noise that are also captured by the questionnaires.

What drives the variability in correlations remains unclear. Studies vary in a number of experimental factors, including hearing aid use, methods of identifying SNRs and details of the administration of the self-report measures. For hearing aid use, inconsistent correlations come from studies where listeners either do not (Heinrich et al., 2015) or do (Ng et al., 2013) wear hearing aids, whereas consistently high correlations were found in a study with a group of listeners with hearing loss where only half of participants used hearing aids (Zekveld et al., 2013). Hence, taking hearing loss into account does not improve the consistency of results. Secondly, the way in which the SNR of behavioral speech perception tests is adjusted (see Heinrich et al., 2015), suggests that procedural details in the measurement of speech perception can affect the correlation with self-report questionnaires. Thirdly, the administration protocol for the self-report questionnaires can affect correlations with speech perception measures, which are higher if the listening situations assessed by self-report and speech perception measures are more closely matched (Ng et al., 2013; Zekveld et al., 2013). Such a practice contrasts with the current practice that typically measures self-report scores as an average across a number of listening situations. This would also contrast with the measurement protocol typically used for behavioral tests, which assess only one situation.

In the present study we also depart from the standard practice of using averaged self-report scores, and instead use each individual listening situation separately for comparison with behavioral intelligibility measures. The main research question is: Does matching specific listening situations between the two different types of measures affect subsequent correlations in hearing aid users with mild-to-moderate hearing loss?

### Speech Perception and Cognition

In addition to the relationship between behavioral and self-report measures of listening, we also sought to better understand the relationship between speech perception and cognition. We have previously tested the predictive power of a number of cognitive abilities for speech perception tests of varying complexity in older listeners with mild hearing loss (Heinrich et al., 2015). We found that cognition only explained a significant amount of variance in speech perception performance in the most complex listening situation, namely sentences presented in modulated noise. A principal component analysis (PCA) was then conducted to extract latent cognitive factors from the multiple cognitive tests used in the study. The PCA produced a two-factor solution with the factors representing working memory (WM) and attention, with only the latent factor of attention showing a predictive value for speech perception. The interpretation of the two factors in the previous paper as WM and attention was guided by Baddeley and Hitch (1974) and Baddeley (2000) who defined WM as the interplay between visuo-spatial and/or verbal information on the one hand and the central executive on the other. Note that the concept of attention is closely related to the concept of executive function (Hasher et al., 2007), and therefore the latent attentional factor in the previous paper might have been more appropriately labeled executive processes. While executive processes are a multifaceted concept and include aspects of attention and inhibition, these facets could not be further differentiated in the previous study due to the small number of cognitive tests, and therefore Baddeley’s model, which united all aspects of attention and inhibition, was the appropriate theoretical framework in that study. However, a model that differentiates executive functions might be more appropriate because executive functions in general, and inhibition in particular (Sommers and Danielson, 1999; Janse, 2012; DiDonato and Surprenant, 2015; Helfer and Jesse, 2015), have been proposed to play a role in complex communication situations (e.g., when communicating in a group where executive function regulates monitoring, attention switching, updating; Ferguson et al., 2014) and in the resultant benefits from auditory training (Ferguson and Henshaw, 2015). Diamond’s (2013) model of executive functions is such a model that articulates a more differentiated view. It also explicitly incorporates Baddeley’s WM component, which was of interest to the current study as WM has been widely suggested to play a role in speech perception (Wingfield and Tun, 2007; Akeroyd, 2008; Mattys et al., 2012). Hence, a second objective of the present study, in addition to assessing the relationship between self-report and behavioral measures, was to assess the contribution of different executive functions and WM to various speech perception tests.

In our previous study (Heinrich et al., 2015), WM tests included digit span (forward and backward), and a visual letter monitoring task, while attention was assessed with single and divided attention tests [Test of Everyday Attention (TEA6 and 7) and the Matrix Reasoning Test]. In the current study, identical or similar tests were chosen to measure WM (Size Comparison Test, Letter Number Sequencing, Dual Digits in Quiet) and attention (TEA6 and 7).

**FIGURE 1:**
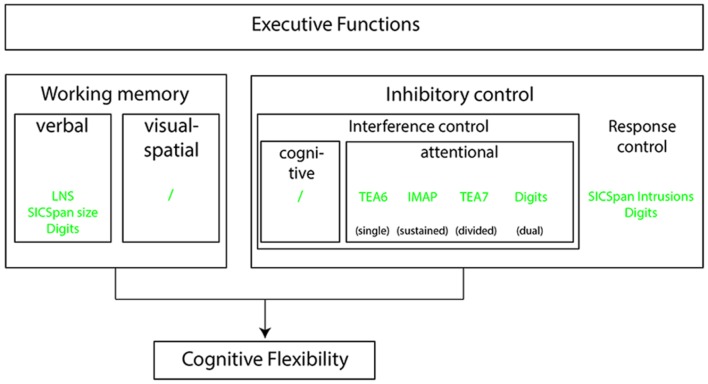
**A schematic of the Diamond (2013) model of executive function.** Also shown are how the study’s cognitive tasks relate to model components. A brief description of the tasks is given in the text, a more detailed description is provided in the method section. LNS, Letter Number Sequencing; SICspan Size, Size Comparison span, span size; Digits, five digit encoding and recall; TEA6/7, Test of Everyday Attention subtests 6 and 7; IMAP, IHR Multicentre study of Auditory Processing test; SICspan Intrusions, Size Comparison span, number of intrusions.

Diamond (2013) distinguishes two different inhibitory control mechanisms, namely interference control and response control. These two control mechanisms differ in the processing stage at which the inhibitory control takes place. Interference control takes place when an individual manages to direct attention away from or suppress a prepotent mental representation. Response control takes place when behavior is controlled despite the urge to follow a prepotent response. Its third core executive function, cognitive flexibility, will not be further discussed. The selection of the remaining cognitive tests was guided by Diamond’s model. How the selected cognitive tests in the current study fit within the model is displayed in **Figure 1**.

Given the large number of cognitive tests, PCA was applied as a data reduction method. PCA, a strictly atheoretical data reduction tool based solely on amount of shared variance between the tests in the analysis, is often the method of choice (e.g., van Rooij and Plomp, 1990; Humes et al., 1994; Schoof and Rosen, 2014; Heinrich et al., 2015). The exploratory PCAs in Heinrich et al. (2015) returned a storage-focussed WM factor and a factor encompassing all other, more attention-focussed processes. When conducting the analysis with comparable tests we predicted that this solution would be replicated. However, on adding the additional tests selected specifically to tests aspects of attention and executive function noted above into the analysis, we predicted that the previous attention factor would be split into two, reflecting the latent variables (i.e., interference and response control) underlying test selection. We also predicted that these three latent cognitive factors would be differentially predictive of speech perception in particular tests.

### Energetic versus Informational Masking

Based on previous research we know that target and background signals differ in the demands they place on cognitive and linguistic processes (Ferguson and Henshaw, 2015; Heinrich et al., 2015), and that it is particularly the complex communication situations that appear to involve executive functions (Ferguson et al., 2014). Therefore, it was important to choose speech stimuli that were sufficiently complex to invoke executive processing. In our previous study, target speech stimuli were phonemes, words and simple sentences, presented either in quiet (the phonemes), or in speech-shaped or white noise (words and sentences). This made perception relatively easy because the maskers were either not present at all or were only energetic in nature (Freyman et al., 1999; Brungart et al., 2001; Arbogast et al., 2002; Kidd et al., 2005).

In the current study, we attempted to increase listening difficulty in two ways: by making the background masker more complex and by presenting one of the speech tasks in a divided attention context. Background masker complexity was increased compared with the previous study by presenting almost all target speech in a speech masker (babble masker or concurrent talker). This extended the previous study by introducing informational masking in addition to energetic masking (Brungart et al., 2001; Arbogast et al., 2002; Kidd et al., 2005; Schneider et al., 2007). For the purpose of the current study, we follow Schneider et al.’s (2007) definition of informational masking as “…any aspect of the background sound that interferes with the processing of the speech signal at more central (cognitive) levels of processing.” In this sense, informational masking should not be viewed as a single phenomenon but rather as resulting from actions at any of the stages of processing beyond the auditory periphery. As a result, it is intimately connected to perceptual grouping and source segregation, attention, memory, and general cognitive processing abilities. As shown by Simpson and Cooke (2005) even high-numbered talker babble can have significant effects of masking above and beyond those provided by speech-shaped noise with the same envelope as the babble. This effect according to Rosen et al. (2013) is driven by the higher similarity between the target speech and babble as opposed to noise. According to Simpson and Cooke (2005) this greater signal similarity between target and background sound for babble may lead to greater attentional demands, greater distracting effects of numerous onsets and general non-stationarity, thus leading to greater masker efficiency.

We also increased the complexity of the speech perception task by adding conditions in which the listening task was not presented in isolation but in concurrence with a memory task. This was intended to increase listening effort. The concept of listening effort is based on Kahneman’s (1973) model of limited processing resources and assumes that performance on a listening task can be affected by the introduction of a second task (e.g., memory), which diverts some of the attention usually available for perception, to another task such as memory encoding. As a result, performance on one or both tasks may decline compared to when the tasks are performed alone (Mattys et al., 2014). The ultimate goal of all these changes in the speech perception tasks was to sample a wide variety of listening situations, and to generally increase the complexity of listening in order to maximize the possibilities of seeing correlations with a range of cognitive functions. Unavoidably, sampling a range of listening situations and changing the characteristics of the foreground and background signal comes at the cost of not being able to investigate systematically which changes in the listening condition cause a change in correlation with self-report and cognition.

### Hearing Loss

A final aspect of listening that was important for this study was the presence of hearing loss and its clinical management with hearing aids. Hearing aids can have tangible consequences not only for the accuracy of listening but also for the involvement of cognitive processes to achieve this (Lunner et al., 2009). Although hearing aids increase the audibility of the signal and thereby might make it easier for the listener to hear the target speech, they also introduce distortions (Edwards, 2007). It has been suggested that adjusting to the unfamiliar and distorted signal requires cognitive input (Arehart et al., 2013). While we cannot directly compare the effect of hearing status on speech perception and self-report between Heinrich et al. (2015) and the current study, as some of the speech perception tests and self-report questionnaires differed, a qualitative comparison was still possible and formed a third aim.

In summary, the current study sought to investigate the relationship between self-report and behavioral speech perception in a group of existing hearing aid users with mild-to-moderate hearing loss. The primary aim was to extend findings about relationships between listening, cognition and self-report from our earlier study in adults with mild hearing loss who did not wear hearing aids, to hearing aid users with mild-to-moderate hearing loss. Based on the previous study we hypothesized the following.

### Hypotheses

H1: As speech intelligibility was not assessed in a way that involved an increase of either background noise or overall stimulus level, we predict no correlation between the speech perception tests and the averaged scores of self-report questionnaires, thus replicating earlier results in those with mild hearing loss. However, when using self-report scores based on specific individual listening situations, we might expect correlations with speech intelligibility scores to emerge when the two listening/speech situations from the self-report and perception test mirrored each other.

H2: We predict the relationship between speech perception and cognition to be not uniform across different speech perception tests but rather to be specific to a particular test, and to become more evident as the complexity of the target speech, defined by its linguistic and other cognitive demands, and the complexity of the masker are increased.

H3: We predict to replicate a two-factor PCA solution with WM storage and attention when including only those tests that are comparable to the previous study. However, when including cognitive tests that include components of executive function, we expect to find a third factor, based on Diamond (2013), that splits the previous attention factor into an interference and a response control executive factor.

## Materials and Methods

The data on which the analyses in this paper are based are the baseline outcome measures of an auditory training study with 50–74 year old hearing aid users with mild-to-moderate hearing loss (Ferguson and Henshaw, 2015). The training task plays no role in the current data. Instead we analyze the outcome measures of speech perception, cognition, and self-report of hearing-related activities at the baseline, pre-training session. The study was approved by the Nottingham Research Ethics Committee and Nottingham University Hospitals NHS Trust Research and Development. Informed signed consent was obtained from all participants.

### Participants

Thirty (20 males) existing hearing aid users (minimum use = 3 months, mean = 10.3 years, *SD* = 10.7 years) aged 50–74 (mean = 67.4 years, *SD* = 7.1) with mild-to-moderate symmetrical sensorineural hearing loss (mean pure-tone hearing thresholds of the better ear averaged across 0.5, 1, 2, 4 kHz = 43.6 dB HL, *SD* = 13.6) were recruited from the NIHR Nottingham Hearing Biomedical Research Unit research volunteer database. Overall, 56% of participants indicated that they used their hearing aids all the time, while 17% used them ≥75% or of the time, and 27% used them 50–75% of the time. All participants spoke English as their first language, and were paid a nominal attendance fee and travel expenses for the visit.

### Procedure

All testing was carried out in a quiet testing room. All auditory stimuli were presented in the free field via a single speaker (Logitech LS 11) situated directly in front of the participant at a distance of 1m, set to individuals’ most comfortable loudness (MCL) level (Ventry et al., 1971), unless otherwise specified. The MCL was set for each participant at the first testing session and kept constant throughout. Participants wore their hearing aids during all testing. Visual stimuli were presented on a 21′ screen (Genelec Inc., Natick, MA, USA) placed 50 cm in front of the participant. Auditory, cognitive and questionnaire responses were obtained in a fixed order, with audiological measures (otoscopy, tympanometry, pure-tone audiometry, MCL) first, followed by speech and cognitive measures in a mixed order that was the same for all participants.

### Outcome Measures

#### Audiological

Outer and middle ear functions were checked by otoscopy and standard clinical tympanometry using a GSI Tympstar (Grason-Stadler, Eden Prairie, MN, USA). *Pure-tone air conduction thresholds* (0.25, 0.5, 1, 2, 3, 4, and 8 kHz) were obtained for each ear, following the procedure recommended by the British Society of Audiology (British Society of Audiology, 2011), using a Siemens (Crawley, West Sussex, UK) Unity PC audiometer, Sennheiser (Hannover, Germany) HDA-200 headphones, and B71 Radioear (New Eagle, PA, USA) transducer in a sound-attenuating booth. The better-ear-average (BEA) across octave frequencies 0.5–4 kHz was derived and is reported here.

#### Speech Perception

The *Phoneme Discrimination (PD)* test (Ferguson et al., 2014) performed in background noise measured the discrimination threshold for one vowel continuum (/e/-/a/) delivered through Sennheiser HD-25 headphones at a fixed level of 75 dBA, presented in 8-Hz modulated speech-shaped noise at 0 dB SNR. The vowel continuum contained 96 steps, which had been synthesized from the real voice recordings at the end points. The continuum was presented in sequential blocks, and all listeners were tested twice. A three-interval, three-alternative forced-choice, oddball paradigm was used. The participant’s task was to choose the odd one out from three sequentially presented phonemes. Feedback (correct/incorrect response) was given. Initially, two (identical) vowel were selected randomly from one end of the continuum and the odd (target) vowel from the opposite end (i.e.,_⋅_wav files #1 and #96). Correct detection of the target, delivered randomly in any of the three intervals, resulted on the next trial in the identical and target phonemes being chosen from a more difficult comparison (e.g., files #11 and #86; i.e., step size 10). Trials then varied adaptively over two, 1-down 1-up reversals, step size 10 and 5, changing to a 3-down 1-up paradigm using a step size of 2 and determining the 79% correct point on the psychometric function (Levitt, 1971). Performance was measured in terms of the separation between stimulus file numbers at threshold. A smaller number signifies better discrimination ability. As the particular vowel continuum here represents a type of phoneme, the resulting threshold was called phoneme discrimination threshold (%), and calculated as the average of the last two reversals over 35 trials.

The *Four Alternative Auditory Feature (FAAF)* test (Foster and Haggard, 1987) assessed phoneme discrimination accuracy in the context of a word in background noise. The overall output level of the stimuli was set at the participant’s MCL for speech. The SNR was fixed at 0 dB SNR. The noise was 20-talker babble noise. The FAAF is a closed-set test with four alternative CVC words per trial. The words vary only in a single phoneme, either the initial (9 sets) or the final (11 sets) consonant of the word. All target words were presented in the carrier sentence “Can you hear ___ clearly” and were followed by the visual presentation of four minimally paired alternatives from which participants chose their response. For instance, the target word *mail* might be paired with *bail*, *nail*, and *dale*. Following a short practice session, 20 test trials were randomly selected from a larger test base and the percentage of correctly perceived words was measured. Responses were given via touch screen and feedback on the correct response was provided.

The *Dual Task of Listening and Memory* required participants to listen to and repeat words while retaining digits in memory. In the speech perception part of the task they listened to lists of five AB isophonetic monosyllabic (CVC) words (Boothroyd, 1968) presented at 65 dB SPL in either quiet or a 20-talker babble background at two SNRs, 0, and -4 dB. Listeners were asked to repeat each word immediately after presentation and were instructed to prioritize both tasks equally. A total of 12 lists (four in each background condition) was presented, with presentation order of noise conditions counter-balanced across participants. A maximum score of 20 per background condition was possible. The word score (Single Words) will be reported as part of the speech perception results.

The *Modified Coordinate Response Measure (MCRM)* (Hazan et al., 2009) measures closed-set keyword perception in a sentence carrier. In contrast to the carrier sentence in the FAAF, of which only one version existed and which was only meant to alert the listener to the presence of the target, the carrier sentence in the MCRM varied in call sign and voice, with only one combination representing the carrier sentence of the target stimulus. The task was based on the Coordinate Response Measure (Bolia et al., 2000). Participants were presented with sentences in the form of ‘Show the [animal] where the [color] [number] is’. There were six possible monosyllabic animals (cat, cow, dog, duck, pig, and sheep), six colors (black, blue, green, pink, red, and white) and eight numbers (1–9, excluding multisyllabic 7). Two sentences were presented concurrently, one by a female talker (target) and one by a male talker (distractor). Participants were asked to listen for the color and number spoken by the female talker (‘dog’ was always the animal target) whilst ignoring the male talker, and to respond by pressing the corresponding target color-number on a computer touchscreen. The test used an adaptive 1-down 1-up staircase method with an initial step size of 10 dB until reversal 1, reducing to 7 dB at reversal 2, and 4 dB at reversal 3 onward and continued until eight reversals were achieved. Speech reception thresholds were calculated as the SNR in dB required to achieve 50% intelligibility in the last two reversals.

#### Self-Report of Hearing Difficulties

The *Glasgow Hearing Aid Benefit Profile* (GHABP) (Gatehouse, 1999) assesses unaided pre-intervention hearing disability (or activity limitations) and handicap (or participation restrictions) in Part 1, and benefit and satisfaction derived from hearing aid (HA), reported HA use, and residual disability (i.e., the disability that remains despite using HA) in Part 2. There are four predefined situations (Q1: Listening to the television with other family or friends when the volume is adjusted to suit other people; Q2: Having a conversation with one other person when there is no background noise, Q3: Carrying on a conversation in a busy street or shop; Q4: Having a conversation with several people in a group), using a five-point scale (residual disability: 1 = no difficulty to 5 = cannot manage at all). The score for each domain was converted to a percentage score. For residual disability, the main communication measure, both the mean overall score averaged across all four situations and the individual scores for each of the four listening situations were considered.

#### Cognitive

Two subtests of the *Test of Everyday Attention* (TEA6 and TEA7) (Robertson et al., 1994) assessed single and divided attention. In the single attention Telephone Search (Subtest 6) participants had to identify 20 pairs of identical symbols, as quickly and accurately as possible, and ignore all other symbols while searching entries in a simulated classified telephone directory. The score was calculated as a quotient between the total time taken to complete the test divided by the number of symbols detected. Lower values represent superior performance. Divided attention was measured with the Telephone Search (Subtest 7, dual task) that was identical to Subtest 6 except participants were additionally required to count and report the number of tones from a string of 1-kHz tones of varying lengths while searching for the symbols. The score was obtained separately for each task, and in combination to give a dual task decrement (DTD). For statistical analyses, the scales for both TEA subtests were reversed to harmonize the direction of scoring with the other cognitive tests where higher scores indicated a better performance.

The *IMAP (IHR Multicentre study of Auditory Processing test)* measures auditory and visual sustained attention by comparing reaction times (RTs) to target stimuli when cues to their presence are either present or absent (Moore et al., 2010). In the auditory modality, listeners were asked to press a button in response to a 1-kHz 200-ms tone presented at 80 dB SPL as quickly as possible. On 20/36 the target sound was preceded by a 125-ms modulated tone with a carrier frequency of 0.6–4.0 kHz and a modulation frequency of 32 Hz, which was presented at 75 dB SPL. Listeners were instructed to regard this “chirp” as a cue to the upcoming target stimulus. In the visual task, participants responded with a button press when an animated character displayed on a computer screen raised their arm. On 20/36 of the trials the arm movement was primed by a change of the character’s t-shirt color. The test comprised a total of 72 trials, 36 auditory and 36 visual, 20 of which in each modality were primed. All targets were spaced 1–4 s apart, and if a cue was present it preceded the target stimulus by 500–1000 ms. In both tests, the mean response times in ms to cued and uncued trials represented the outcome variable.

The *Letter Number Sequencing (LNS)* task (Wechsler, 1997) is a measure of verbal WM in which participants were asked to repeat a string of pre-recorded numbers and letters (e.g., 4-S-6-A) with numbers in numerical order first, followed by letters in alphabetical order (e.g., 4-6-A-S). Sequences began with two items and had the potential to increase to a maximum of eight items. For each sequence length, three trials were presented for which a participant needed to correctly recall at least one out of the first two trials in order to advance to the third trial and the next longer sequence. When no trial of a particular sequence size was correctly recalled, the task was terminated. The overall number of correctly recalled sequences was used as outcome measure.

The *Size Comparison Span (SICspan)* (Sörqvist et al., 2010) measures the ability to exclude irrelevant information from WM while retaining target items for later recall, thus testing verbal WM together with aspects of response inhibition. The task consisted of two parts, a size judgment task and a memory task. The stimuli to the first task were to be ignored after the task was completed. For instance, participants were presented with the following words: “Is CAT larger than COW? CROCODILE,” were expected to respond yes or no to the size comparison element (in this example no) and then encode the third word into memory (i.e., crocodile) for recall at the end of the list. The total number (out of 40) of correctly recalled memory words (SICspan Size) was the outcome measure. When a participant recalled a size comparison words instead of a target word, this was classed as a list intrusion (SICspan Intrusions). The total number of intrusions (out of a possible 80) was summed across the whole test.

The *Dual Task of Listening and Memory* required participants to listen to and repeat words while retaining digits in memory, and was originally designed to assess listening effort (Howard et al., 2010). Participants were asked to encode a string of five digits displayed on a computer screen during a 5 s period for later recall. After encoding, listeners completed the speech perception task as described above. After the completion of the speech perception task participants were asked to recall the encoded digits. A maximum score of 20 was possible per noise condition. In the following the digit score (Digits) will be reported as part of the cognitive results.

## Results

**Table 1** displays the descriptive information for all variables of interest in the study. Note that the perception component of the Dual Task (Words) is classified as a word perception task while the memory component of the same task is classified as cognitive task (Digits), even though both elements of the task were always presented concurrently, in three different background noise conditions.

**Table 1:** Mean, standard deviation (SD) and range for demographic information and experimental variables.

				Mean	*SD*	Range
Demographic information	Age (years)			67.4	7.1	50.0–74.0
	Better ear average (dB HL), BEA	4 freq (0.5, 1, 2, 4 kHz)		43.6	13.6	25.0–77.5

	**Speech task**	**Target speech type**	**Masker type**			

Speech perception	Discrimination of isolated phonemes (step size)	PD	8-Hz modulated noise at 0 dB SNR	74.2	12.1	54–99
	Discrimination of phonemes in words (percent correct)	FAAF	20-talker babble at 0 dB SNR	63.5	12.8	37.7–82.4
	Word perception (number correct/20)	Words	Quiet	15.9	3.7	6.0–20.0
		Words	20-talker babble at 0 dB SNR	5.9	3.2	0.0–13.0
		Words	20-talker babble at – 4dB SNR	2.2	2.7	0.0–13.0
	Keyword perception in a carrier sentence (dB SNR)	MCRM	single-voice at variable SNR	-4.3	5.6	-15.0–+5.0

	**Cognitive domain**	**Task**				

Cognition	Single attention (time for completion / no. correct)	Test of everyday attention (#6), TEA6		3.5	0.6	2.4–5.0
	Divided attention (time for completion / no. correct)	Test of everyday attention (#7), TEA7		3.9	1.1	2.4–6.6
	Attention-related decrement	Test of everyday attention (Dual task decrement), DTD		1.1	1.6	-0.5–6.8
	Verbal WM under dual attention (number correct/20)	Digits in quiet		15.2	4.3	5.0–20.0
		Digits at 0 dB SNR		12.1	4.6	5.0–20.0
		Digits at -4 dB SNR		14.6	5.1	0.0–20.0
	Sustained attention (reaction time in ms)	IMAP Visual uncued		427.2	123.7	268.3–922.6
		IMAP Visual cued		330.1	91.1	234.1–640.7
		IMAP Visual difference		97.1	75.4	-34.0–281.8
		IMAP Audio uncued		597.7	156.3	289.3–1033.9
		IMAP Audio cued		401.5	124.7	226.6–880.0
		IMAP Audio difference		196.3	98.3	0.0–375.4
	Verbal WM and response control (number correct/40)	SICspan Size		29.0	5.1	8.0–35.0
	(number correct/80)	SICspan Intrusions		3.4	1.7	1–8
	Verbal WM (number correct/24)	Letter Number Sequencing (LNS)		9.2	2.5	4.0–17.0

Self-report	GHABP (Residual Disability only) (percent)	Overall		27.5	15.0	0.0–56.25
		Q1: Listening to TV		25.0	23.7	0.0–75.0
		Q2: Conversation in quiet		12.5	15.7	0.0–50.0
		Q3: Conversation in a busy street / shop		28.3	22.5	0.0–75.0
		Q4: Group conversation with several people		44.2	21.5	0.0–75.0

*PD, Phoneme Discrimination task; FAAF, Four Alternative Auditory Feature word perception task; Words, single word perception aspect of dual task; MCRM, Modified Coordinate Response Measure; Digits, five digit encoding and recall aspect of Dual task with word perception task presented in quiet, at 0 dB SNR, -4 dB SNR; IMAP, IHR Multicentre study of Auditory Processing test; SICspan Size, Size Comparison span, span size; GHABP, Glasgow Hearing Aid Benefit Profile.*

**FIGURE 2:**
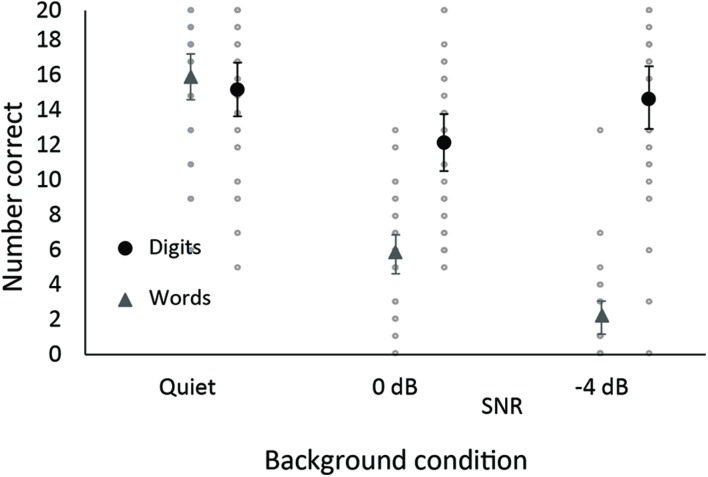
**Means (and 95% confidence interval) for single word perception (triangle) and digit memory (circle) in the same dual task.** Individual data are displayed as faint gray circles.

For the Dual Task of listening and memory there was a steady decline in the intelligibility of the words (Words) from the quiet condition to the 0 dB SNR to the -4 dB SNR condition (**Figure 2**). In contrast, memory for the digits in the same task (Digits) showed a decrease from the quiet to 0 dB SNR condition, but recovered at the most adverse noise level (-4 dB SNR). Two repeated-measures ANOVAs for Words and Digits with background condition (quiet, 0 dB SNR, -4 dB SNR) as the only factor confirmed these patterns (Words: *F*[2,58] = 238.5, MSE = 6.3, *p* < 0.001, Quiet > 0 dB SNR > -4 dB SNR; Digits: *F*[2,58] = 5.77, MSE = 13.6, *p* = 0.005, Quiet = -4 dB SNR > 0 dB SNR).

The IMAP attention task showed that RTs to cued stimuli were faster than to uncued stimuli, that RTs to visual stimuli were generally faster than to auditory stimuli, and that the difference between cued and uncued stimuli was greater for visual than auditory stimuli (**Table 1**). These patterns were confirmed in a 2 modality (visual, auditory) × 2 cue (no cued, cued) repeated-measures ANOVA, which showed main effects for modality (*F*[1,29] = 64.2, MSE = 6837.5, *p* < 0.001), and cue (*F*[1,29] = 126.9, MSE = 5086.3, *p* < 0.001) and an interaction between the two (*F*[1,29] = 28.4, MSE = 2593.2, *p* < 0.001).

### Correlation between Speech Perception Tests and Self-reported Communication Abilities

H1: As speech intelligibility was not assessed in a way that involved an increase of either background noise or overall stimulus level, we predict no correlation between the speech perception tests and the averaged scores of self-report questionnaires.

Pearson Product-Moment correlations between the overall score of residual disability and speech perception were as follows: Phoneme Discrimination (PD) in noise *r* = -0.42 (*p* = 0.03); FAAF *r* = -0.26 (ns); word perception dual task (Words) in quiet *r* = -0.26 (ns), 0 dB SNR *r* = -0.38 (*p* = 0.04), and -4 dB SNR *r* = -0.29 (ns), MCRM *r* = 0.29 (ns). Hence, there were two significant correlations with the overall residual disability score: with PD and with Word perception at 0 dB SNR. For Word perception this means that listeners with better intelligibility scores tended to have lower residual disability scores, as might be expected. When BEA was partialled out, the correlation disappeared (*r* = -0.12). The correlation between PD scores and residual disability was both unexpected and counterintuitive and was unaffected by hearing loss (*r* = -0.47 with BEA partialled out), and suggested that listeners with better phoneme discrimination ability (lower scores) tend to have higher disability scores. We speculate about the underlying reasons for this result in the Discussion section.

More pertinent, however, are the correlations between the speech perception tests and the residual disability score for each of four individual GHABP situations, shown in **Table 2**.

**Table 2:** Spearman correlation coefficients between residual disability scores for each of the four GHABP listening situations and the speech perception tests.

	Phonemes	Isolated Words				Words in Sentences
	Discrimination in Noise	FAAF	Words			MCRM
			Quiet	0 dB SNR	-4 dB SNR	
Listening to TV (Q1)	-0.45*	-0.15	-0.11	-0.33	-0.35	0.17
Conversation in quiet (Q2)	0.08	0.05	-0.02	-0.19	0.14	0.05
Conversation in a busy street/shop (Q3)	-0.24	-0.41*	-0.24	-0.54**	-0.36*	0.26
Group conversation with several people (Q4)	-0.41*	-0.17	-0.11	-0.08	-0.02	0.41*

Acronyms as in **Table 1**. *p < 0.05, **p < 0.01.

Spearman coefficients were used because of the ordinal scale on the GHABP. Except for listening to a conversation in quiet (i.e., the easiest listening situation), all the GHABP pre-defined situations were significantly correlated with performance on at least one speech perception test. Listening to a TV set to someone else’s need (Q1) correlated with PD, following a conversation in a busy street or shop (Q3) correlated with performance on word perception tests in noise (i.e., FAAF and single word perception), and following a group conversation with several people (Q4) correlated with performance on both PD and the MCRM keywords in the carrier sentence. Similar to the overall score results, correlations between word perception and residual disability were in the expected direction with better speech performance scores associated with lower residual disability scores for Q3 (conversation in a busy street). Unlike the overall scores, it was not only Word perception at 0 dB SNR that showed a significant correlation to the self-report residual disability score, but also Word perception at -4 dB SNR and the FAAF. All of these tests require listeners to perceive words in a background of noise.

### Correlation between Speech Perception and Cognition

H2: We predict the relationship between speech perception and cognition to be not uniform across different speech perception tests but rather to be specific to a particular test, and to become more evident as the complexity of the target speech, defined by its linguistic and other cognitive demands, and the complexity of the masker are increased.

There were moderate, significant correlations for BEA with all the speech perception tests except PD in noise and Word perception at -4 dB SNR (see Supplemental Information). The correlation was negative for FAAF and Word perception reflecting the fact that increased BEA thresholds were associated with decreased perceptual accuracy. The correlation was positive for MCRM because an increased BEA was associated with an increased SNR. PD did not correlate with performance on any cognitive test, whereas performance on the MCRM correlated with performance on a broad range of cognitive tests. Performance on the FAAF and Word perception tests were most strongly correlated with verbal WM (LNS), alongside a correlation with one other cognitive test each. Exact values for all correlations are reported in Supplementary Table S1. Because most speech perception tests significantly correlated with BEA, Supplementary Table S2 presents the same correlations with BEA partialled out. The correlational patterns did not change substantially when hearing sensitivity (BEA) was partialled out. These patterns are consistent with our previous study (Heinrich et al., 2015) in that there were different cognitive profiles for different speech perception tests. Self-reported residual disability correlated with cognition in only two instances, but notably these occurred for the two situations (conversation in a busy shop, conversation with a group of people) that are most likely to engage cognition (Supplementary Table S3).

### Latent-Factor Analyses (PCA)

H3: We predict to replicate a two-factor PCA solution with WM storage and attention when including only those tests that are comparable to the previous study. However, when including cognitive tests that include components of executive function, we expect to find a third factor, based on Diamond (2013), that splits the previous attention factor into an interference and a response control executive factor.

Cognitive tests that were broadly comparable between the current and the previous study were TEA6/7, LNS, SICspan Size, and the Digits in quiet. The two TEA tests were identical between the two studies. LNS and SICspan Size tests were similar to the Backward Digit Span (BDS) and Visual Letter Monitoring (VLM) of the previous study as all four tests are WM tasks with storage and processing components. The LNS was deemed particularly similar to BDS and VLM tasks because in all of these tasks the processing component was integral to the span task. In contrast, the SICspan Size test contained a processing component that was not integral to completing the span task. The Digit Quiet task was included because it was also similar to the Digit Span Forward task: both were pure serial recall/storage tasks without a processing component. Using these five cognitive tests in a PCA with Varimax Rotation that extracted all factors with eigenvalues > 1 led to a two-factor solution that explained 64.9% of the overall variance (KMO = 0.5, Bartlett’s test of sphericity: χ^2^(10) = 28.1, *p* = 0.002). Factor loadings are displayed in **Table 3**.

**Table 3:** Factor loadings for five cognitive tests producing a two-factor solution in a Principal Component Analysis.

	Attention (36.2%)	Working memory (28.7%)
TEA6	0.93	-0.03
TEA7	0.84	-0.07
SICspan Size	0.01	0.69
LNS	-0.49	0.60
Digit Quiet	-0.05	0.78

*Acronyms as in **Table 1**.*

The solution replicated the factor structure found in our previous study (Heinrich et al., 2015) in that it showed an attentional factor on which the Tests of Everyday Attention (TEA) loaded and a WM factor on which the tasks with storage and processing components loaded. A second analysis included all the cognitive tests, which had been selected to specifically assess executive function: sustained attention (IMAP visual, IMAP audio), aspects of inhibitory control (SICspan Intrusions, Digits), and dual attention (Digits at 0 and -4 SNR). Note that for tests with measures for individual subcomponents as well as difference scores, such as the TEA and IMAP, only one or the other was included in the PCA. For the TEA, the two component tests TEA6 (single attention) and TEA7 (divided attention) but not their difference score was included. This was done in order to preserve continuity to the previous study which had also included the component scores into the PCA. For the auditory and visual IMAP tests, only the difference scores were included because no precedence for using the component scores existed, and using the difference scores was a more efficient way of combining information. A PCA with Varimax rotation that extracted all factor eigenvalues > 1 resulted in a three-factor solution that explained 63.3% of overall variance (KMO = 0.6, Bartlett’s test of sphericity: χ^2^(45) = 86.0, *p* < 0.001). Factor loadings are displayed in **Table 4**.

**Table 4:** Factor loadings for five cognitive tests producing a three-factor solution in a Principal Component Analysis.

	Factor 1 (27.3%)	Factor 2 (20.8%)	Factor 3 (15.3%)
TEA6	0.81	0.02	-0.37
TEA7	0.73	0.07	-0.34
SICspan Size	0.02	0.16	0.55
LNS	-0.22	0.08	0.77
Digit Quiet	0.00	0.78	0.37
Digit 0 dB SNR	-0.31	0.71	0.14
Digit -4 dB SNR	-0.71	0.42	0.00
SICspan Intrusions	0.26	0.78	-0.09
IMAP visual difference	0.64	0.34	0.13
IMAP audio difference	0.63	-0.15	0.46
Interpretation	Attentional interference control	Response control	Verbal WM

*Acronyms as in **Table 1**.*

Some aspects of the 3-factor model looked similar to the 2-factor model, even though factor labels have changed. TEA subtests loaded on one component, while SICspan Size and LNS loaded on another. The most notable change between the two models was that the storage factor loading of Digit Quiet was less pronounced. Instead, Digit Quiet, together with Digit 0 dB SNR (and to some extent Digit -4 dB SNR), loaded on a new factor (i.e., not WM). Factor labels reflect to some extent constructs of the Diamond (2013) model. A high score on Factor 1 combines good performance on TEA tests, a large difference between uncued and cued attentional IMAP trials and poor Digit memory at -4 dB SNR. The factor may indicate the involvement of attention (as indexed by TEA scores), but also an inability for sustaining attention and inhibiting extraneous distractors. As such it may be indicative of poor attentional interference control. A high score on Factor 2 that combines good Digit memory in quiet and at 0 dB SNR (and to some extent at -4 dB SNR) with many intrusion errors on the SICspan task, may indicate a good memory storage enabling good memory performance despite intrusion of other information. A high score of Factor 3 that combines SICspan Size and LNS, indicates good verbal WM processing performance. The importance of the processing component for this factor might be emphasized by the fact that the IMAP difference score also has a secondary loading on this factor. Note that all principal component analyses are *post hoc* and exploratory as factor extraction is solely based on the amount of shared variance between measured tests. The resultant factor structure is therefore not theoretically motivated and should be interpreted with caution.

In a final analysis we investigated the effectiveness of the three latent factors for the prediction of speech perception performance and self-reported residual disability (**Table 5**). Forward stepwise regression analyses on the six speech perception tests were performed. BEA and age were always entered in a first step, all latent factors were entered together in the second step.

**Table 5:** Results for forward stepwise regression models carried out for each of six speech perception tests.

Speech test	Step	*r*	*r*^2^	Adj *r*^2^	*SE*	*r*^2^ change	*F* change	df_1_	df_2_	Sign.	sign. predictors
Phoneme	1	0.43	0.18	0.12	22.76	0.18	2.93	2	26	0.071	
	2	0.44	0.19	0.01	24.12	0.01	0.05	3	23	0.985	

FAAF	1	0.69	0.48	0.44	9.60	0.48	12.23	2	27	0.001	BEA
	2	0.86	0.74	0.68	7.18	0.26	8.07	3	24	0.001	verbal WM

Words Quiet	1	0.73	0.53	0.50	2.62	0.53	15.48	2	27	0.001	BEA
	2	0.77	0.59	0.50	2.61	0.05	1.01	3	24	0.404	

Words 0 dB SNR	1	0.66	0.43	0.39	2.47	0.43	10.16	2	27	0.001	BEA
	2	0.78	0.60	0.52	2.19	0.17	3.50	3	24	0.031	verbal WM

Words -4 dB SNR	1	0.25	0.06	-0.01	2.69	0.06	0.87	2	27	0.432	
	2	0.60	0.35	0.22	2.36	0.29	3.64	3	24	0.027	verbal WM

MCRM	1	0.56	0.32	0.27	4.79	0.32	6.27	2	27	0.006	BEA
	2	0.77	0.59	0.51	3.93	0.27	5.35	3	24	0.006	response control, verbal WM

Q1	1	0.46	0.21	0.16	21.76	0.21	3.67	2	27	0.039	BEA
	2	0.49	0.24	0.08	22.75	0.02	0.23	3	24	0.873	

Q2	1	0.26	0.07	0.001	15.75	0.07	0.99	2	27	0.384	
	2	0.40	0.16	-0.01	15.83	0.09	0.90	3	24	0.455	

Q3	1	0.35	0.12	0.06	21.82	0.12	1.90	2	27	0.169	
	2	0.52	0.27	0.12	21.12	0.15	1.61	3	24	0.214	

Q4	1	0.41	0.17	0.11	20.28	0.17	2.73	2	27	0.083	
	2	0.42	0.17	0.01	21.45	0.01	0.05	3	24	0.985	

*In step 1 age and BEA was added. In step 2 the three latent cognitive factors (see **Table 5**) were entered. Acronyms as in **Table 1**.*

For PD in noise, cognition did not predict performance. For all other speech perception tests (FAAF, Words, MCRM), cognition predicted performance. For all the Word perception tests, the verbal WM component drove the predictive power of cognition. For the MCRM task, in addition to verbal WM, response control and to a lesser degree attentional interference control also contributed to explaining variance. The cognitive test that probably drove the predictive power of the verbal WM component was the LNS, which showed correlations with all word-in-noise tests (Supplementary Tables S1 and S2). Consistent with the fact that MCRM performance was predicted by a broader range of cognitive components is the finding that it was correlated with a broader range of cognitive tests (Supplementary Tables S1 and S2). Interestingly, even though both Q3 and Q4 each correlated with one cognitive measure (Supplementary Table S3), this was not reflected in the regression analysis after the measures had been combined into latent variables. For instance, there was a moderate correlation between Q3 and the IMAP auditory difference score. However, this difference score was only one of five scores that formed the attentional interference score, and indeed it only had a loading of 0.63 on the latent factor. Very likely, the correlation was not strong enough to overcome its small role on the latent factor.

## Discussion

It is common for many older adults to find it challenging to communicate effectively in noisy environments. The discomfort and frustration resulting from this can prompt withdrawal or avoidance of social situations, which can in turn severely limit activities (Heffernan et al., 2016). This can result in a less active and satisfying lifestyle, and may lead to depression (Cohen-Mansfield et al., 2010; Mikkola et al., 2016). Understanding why older listeners struggle with speech perception in noisy situations is a critical first step to any rehabilitative effort to ensure successful communication, active aging and well-being. One vital question in this context is how to best measure communicative functioning. Self-report and behavioral measures are widely used. Intriguingly, these measures seem to provide information that can seem contradictory, as self-reported difficulties are not always captured by behavioral tests, and behavioral test results do not always reflect listener experience. A better understanding of why the results of these two types of measures are so poorly correlated may guide us to construct speech-in-noise tests that better reflect the listener’s everyday experience, which would provide a first step to successful rehabilitation.

Here, we approached this question from two perspectives. First, we investigated whether we could better understand the relationship between self-report and behavioral tests by being more specific about individual listening situations, both behavioral and self-report. Hence, we investigated the association between behavioral speech perception tests and specific self-report situations rather than just the averaged overall scores of questionnaires.

Second, we investigated the role of cognition in the understanding of listening difficulties. It has long been known that cognition is important for speech perception (Akeroyd, 2008), but which cognitive aspects support listening in which situation, remains to be understood. Recently, we have argued that the relationship between speech perception and cognition is specific to the particular speech test condition (Heinrich et al., 2015), and that more complex listening situations engage more and different aspects of cognition than less complex listening situations (Ferguson et al., 2014). Here, we expanded on this notion by considering a range of specific listening situations, from simple (phonemes in modulated noise) to complex (keyword perception in a carrier sentence with competing talker), and a greater range of theoretically motivated cognitive functions than previously (Heinrich et al., 2015).

In addition to the relationship between behavioral tests, self-report measures and cognition, it is also important to bear in mind that listeners’ sensory auditory function declines as they age and that they have increasing difficulties with listening to speech in noise (CHABA, 1988; Pichora-Fuller, 1997). While auditory decline and speech-in-noise perceptual difficulties are related to some degree, the relationship is far from perfect (Luterman et al., 1966; Phillips et al., 2000; Schneider and Pichora-Fuller, 2001; Pichora-Fuller and Souza, 2003; Gifford et al., 2007). In our earlier paper, we investigated older adults with mild hearing loss who had not sought hearing aids. In the current paper we expanded the range of participants to older listeners with a mild-to-moderate hearing loss who wore hearing aids. We investigated whether the previously found relationships in Heinrich et al. (2015) would hold for a group of listeners who used hearing aids (the current study). One aspect that remained similar across studies was the nature of the target speech; both studies used single CVC words (digit triplet test vs. FAAF test and single word perception), and either a simple sentence or a keyword in a carrier sentence measure (Adaptive Sentence List vs. MCRM). However, our two studies were not directly comparable in quantitative terms as some test measures, particularly background maskers and cognitive tests, had changed. Lastly, because the hearing sensitivity characteristics of the listeners had changed, the most appropriate aspect of self-report assessed in the GHABP changed from initial disability (used for non-hearing aid users) to residual disability after hearing aid use. Nevertheless, both studies tested similar concepts (speech perception in noise; self-report; cognition) and thus are comparable in principle. Specific hypotheses are discussed below.

### Correlation between Speech Perception Tests and Self-Reported Communication Abilities

The first hypothesis concerned correlations between speech perception accuracy and overall scores of self-reported hearing disability. Heinrich et al. (2015) failed to find significant correlations for the vast majority of comparisons in which speech perception was assessed without raising the overall presentation level. We replicated the failure to find consistent correlations between speech perception and overall self-report scores. Only for two of the speech perception tests did the overall GHABP residual disability score correlate with speech perception. Those tests were Word perception in quiet and Phoneme Discrimination (PD) in noise. Moreover, the correlation was in the expected direction only for the former test where better perception scores correlated with lower perceived disability. The correlation with PD was counterintuitive; we can only speculate as to why this happened. Possibly, listeners who function well in their auditory environments and in psychometric speech perception tests employed a very different listening strategy for PD task compared to listeners who generally function less well in auditory environments. A direct comparison with the previous study, which found no correlation at all, is made difficult by the fact that the PD task had been previously presented in quiet whereas here it was presented in noise. One potentially interesting detail is that if one considers the correlation sizes in the studies between behavioral measures of speech perception and overall self-report scores it was only the correlation involving PD that was significantly higher in the current than the previous study; all other correlation coefficients were roughly of a similar size.

In contrast to a relative lack of significant correlations with overall self-report scores, some consistent patterns emerged for correlations between specific GHABP situation scores and each speech perception test. For instance, there were consistent significant correlations between the situation describing a conversation in a noisy background (Q3) and all but one (Words in Quiet) word perception in noise tests. The tests with significant correlations (FAAF and Word perception at 0 and –4 dB SNR) all shared two features that distinguished them from all other tests: first they required listeners to perceive isolated words, second all words were embedded in 20-talker babble. The consistent correlations suggest that either or both of these characteristics assess an aspect of listening that is also important for following a conversation in noise (Q3). It also suggests that this aspect is not assessed by either PD in noise or by word perception in a carrier sentence masked by a single talker (MCRM).

The correlations between cognition and speech perception tests suggest that performance on word perception tests covaries mainly with verbal WM (LNS). This is in agreement with Akeroyd (2008) who found that results in most of the speech-in-noise perception studies surveyed correlated with verbal WM. Why this correlation only occurred with LNS but not SICspan Size is a matter of speculation. One possible interpretation is that a WM task only measures skills relevant to speech-in-noise perception when the task involves the manipulation of the recalled material (as is the case in the LNS task) and not when the manipulation and recall concerns separate materials (as in the case of the SICspan Size). Something in the listening task, either the separation of words from background noise or the dealing with multi-talker babble, uniquely engages verbal WM as measured by the LNS task. The same aspect of the LNS task may also provide the link to the self-report measure. While the correlation between LNS and Q3 is not significant, numerically it does provide the second highest value of correlations between Q3 and cognition, lending at least some credence to our speculation.

Finally, the MCRM task engaged more cognitive processing than solely LNS, possibly diluting any correlation with the self-report ratings on Q3. Instead, performance on MCRM sentences correlated with self-reported functioning in a more complex situation (i.e., participating in a group conversation, Q4) where more complex speech phrases and lower number background talkers are more common. While the correlation between MCRM and group conversation (Q4) makes intuitive sense, it is less intuitive to understand why self-rated ability to hold a group conversation also shares common variance with PD in noise. We speculate that this result may be an expression in the use of different listening strategies between listeners who functioned well or not so well in their auditory environments and in psychometric speech perception tests. PD in noise also correlated with self-reported residual disability concerning the TV level set to suit other people’s need (Q1). In both cases, Q1 and Q4, the correlation with PD was negative indicating that better self-reported functioning in the listening situation was associated with worse performance on the PD test.

The current data set cannot differentiate between the two interpretations of whether it was the foreground speech (words as opposed to phonemes or phrases) or the background (multi-talker babble as opposed to modulated noise or single talker background) that led to the distinct correlations with cognitive processing and self-report. This question will have to be addressed in a future study that manipulates the characteristics of the background sound systematically.

Self-rated residual disability in our group of hearing aid users was largely independent of cognitive ability. Only the questions from the more challenging situations (following a conversation on a busy street (Q3), and in a group of several people (Q4)) showed a single correlation with cognition. In both cases this was either cued attention, or the difference between cued and uncued attention, presumably because for the situations described in Q3 and Q4, an ability to be able to pay attention is crucial to successful listening. However, neither correlation was strong enough to be a significant predictor in the regression model of latent predictors. These differences in correlations with cognitive abilities between behaviorally measured speech perception and self-reported residual disability implies that the correlation shared between word perception tests and Q3, and MCRM and Q4, was not moderated by cognition alone, and must reflect some other shared dimension between these measures.

However, there is also a more conceptual difference between behavioral speech perception tests and self-report measures, which may explain some inconsistencies in correlations and which is much more difficult to tackle. In particular, it is possible that many laboratory-based behavioral speech perception tests do not capture the demands of listening in the real world. Examples for mismatches between the two types of situations are the fact that the SNRs in laboratory-based speech perception tests are often more adverse than in real life situations (Smeds et al., 2015), that they often neglect reverberation and that they are often perception or comprehension tests without the necessity for the listener to engage in two-way interactions. If listeners refer to memories of their real life situations when responding to self-report measures, then it is not surprising that correlations between behavioral speech perception tests and self-report measures are often low. In order to remedy this issue, the conceptualization of speech perception tests as a whole would need to be reassessed.

### Correlation between Speech Perception and Cognition

We found no significant correlations between cognitive performance and PD suggesting that none of the cognitive abilities tested here (attentional interference control, response control, verbal WM) played a role in this speech task. This replicates previous null findings from Heinrich et al. (2015). All other speech perception tests correlated highly with the LNS suggesting that good verbal WM abilities were important for good performance on these speech perception tests. As seen from the correlations (Supplementary Tables S1 and S2) but not the latent-variable regressions, each speech test was also associated with an additional specific cognitive ability, which varied from test to test. For the FAAF it was the SICspan Size test that measured the ability to exclude irrelevant information from WM while retaining target information for later recall. For Word perception it was either sustained attention when words were presented in quiet or good memory storage when words were presented at an adverse SNR. Presumably in the former case, good performance mostly depended in being able to keep attention on perception (while also holding digits in memory) in order to hear all the words, while in the latter, a good memory helped because it meant that less attentional resources were needed to retain the digits in memory and more resources could be spent on the speech perception task. The MCRM task engaged a wide variety of cognitive abilities. Taking these cognitive profiles into account when interpreting speech performance may help us understand why some listeners do better than others and how we can choose speech perception tests that either maximize or minimize cognitive differences between listeners.

In showing different cognitive profiles for different speech perception tests the study replicates a main finding from Heinrich et al. (2015), despite a different group of listeners and slightly changed speech perception and cognitive tests. This replication under changed circumstances suggests that speech perception tasks do indeed differ in cognitive profile and that the previous results were not due to the peculiarities of either the listener group or the combination between speech and cognitive tests. Different cognitive profiles for different listening situations have also been found by Helfer and Freyman (2014). The finding also extends a number of previous studies that either used one speech perception test and a number of cognitive tests (Besser et al., 2012; Zekveld et al., 2013) or a number of speech tests and only one or two cognitive tests (Desjardins and Doherty, 2013). Until now, no systematic comparison between speech perception situations and cognitive abilities exists across a range of systematically varied listening situations, and comparisons always have to be made across studies. These direct comparisons between studies are difficult as typically both fore- and background sounds as well as the assessed cognitive abilities change from study to study.

A much more systematic and theoretically driven approach to the variation of fore-and background speech as well as the assessment of cognitive function is needed. With this and the previous (Heinrich et al., 2015) study we are attempting to start laying the ground for such theoretical underpinnings by discussing selected cognitive tests within wider frameworks of cognitive functioning (see the third hypothesis). In the previous study we discussed the cognitive results within latent factors for WM storage and attention, considered within the framework of Baddeleys WM model of storage and manipulation (Baddeley and Hitch, 1974; Baddeley, 2000). We concluded that manipulation (attention) was particularly important in the most complex listening situation. Here, we expanded on the notion of attention by putting it within the framework of executive functioning as represented by Diamond (2013), a concept that has long been claimed to be important for speech perception (Sommers and Danielson, 1999; Janse, 2012; DiDonato and Surprenant, 2015; Helfer and Jesse, 2015). In using a more differentiated approach to testing executive functions that considers executive functions, not as a whole (as does Baddeley), but as distinguishable processes (cognitive and attentional interference control, response control), we were able to specify that it may have been the response control aspect of attention that predicts speech perception performance in more complex listening situations.

One difference we found between Heinrich et al. (2015) and the current study is the fact that single word perception, operationalized as triple digits in the previous study and as FAAF or single words in a dual task in the current study (Words), seemed to engage different cognitive processes. Whereas triple digits performance was not predicted from the cognitive performance of the tests assessing WM and attention, FAAF and Word perception was predictable from the WM performance as measured here. Two possible explanations for this divergence in results are considered. First, the Word perception task in the current study might have been more complex. The pool of target words consisted of more than a closed set of nine digits, and the background sound was multi-talker babble not speech-shaped noise. Maybe this difference was enough to engage WM. Alternatively; it is possible that the change in listener group caused the change in correlational pattern. While single word perception operationalized as digits in noise does not engage WM in older listeners with no hearing aids, it does so in older hearing aid wearers. Differentiating between these two interpretations requires a direct comparison between these listener groups within the same study.

The current study was explorative in nature, set up to test the influence of a large range of possible variables. As a result, test selection of both speech perception tests and cognitive tasks was not as systematic as a careful elucidation of mechanisms would have demanded. On the other hand, however, this approach allowed us to define conditions that should be satisfied in future studies in order to advance our understanding of cognitive contributions to speech perception. First, listening situations need to be more complex than perception of single words, in order to draw out executive contributions. Second, the characteristics of fore- and background signals need to be systematically and parametrically manipulated to understand which aspect of listening engages which aspect of cognition. Third, embedding cognitive test selection within general cognitive frameworks may allow us to discuss cognitive processes not only on the level of selected tests but on the level of underlying cognitive components, and may thus make it easier to compare across studies. It may also allow us to connect speech perception research more closely with the wider cognitive research community.

There were some limitations of study design and analysis, which restricted the interpretability of the results. First, both target speech and maskers were varied across conditions, which made an interpretation of the results concerning the associations with self-report measures and cognition harder and less reliable. Moreover, although in agreement with other recent studies (Corbera et al., 2013; Schoof and Rosen, 2014; Heinrich et al., 2015), the study sample was small for the number of statistical analyses. This increases the risk of false positive findings. Only by repeating the study with independent groups of participants and by using larger sample sizes will it be possible to establish which correlations are replicable.

## Conclusion

These exploratory results replicate and extend our previous findings by investigating the relationship between a set of speech perception tests and cognitive measures, which were more complex, in aided listeners with mild-to-moderate hearing loss. We found that the association between speech perception performance and cognition varied with the specific tests used, showed that verbal WM in particular appears to be important for the speech perception tests used, and that correlations were evident when behavioral speech perception tests and listening situations in self-report questionnaires matched in some characteristics. Finally, cognition did not correlate with self-report of communication. The next step is to test these conclusions with systematic hypothesis-driven research.

## Author Contributions

MF and HH designed the study. AH analyzed and interpreted the data. AH wrote, and MF contributed to, the manuscript. AH and MF contributed to critical discussions. AH and MF revised the manuscript. All authors approved the final version of the manuscript for publication. All authors agree to be accountable for all aspects of the work and in ensuring that questions related to the accuracy or integrity of any part of the work are appropriately investigated and resolved.

## Conflict of Interest Statement

The authors declare that the research was conducted in the absence of any commercial or financial relationships that could be construed as a potential conflict of interest.

## Footnotes

1 Non-hearing aid users here and elsewhere refers to people who have not been prescribed and issued with hearing aids.
