# Supplementary material for: Only Behavioral But Not Self-Report Measures of Speech Perception Correlate with Cognitive Abilities
Source: Front Psychol. 2016 May 23;7:576. doi: 10.3389/fpsyg.2016.00576 (PMC4876806; doi:10.3389/fpsyg.2016.00576)
Supplement: Supplementary file 1 [file Data_Sheet_1.PDF]

Supplementary Material

Only behavioural but not self-report measures of speech perception correlate with cognitive abilities

Antje Heinrich[1]*, Helen Henshaw[2], Melanie Ferguson[2,3]
[1]MRC Institute of Hearing Research, Nottingham, UK
[2]NIHR Nottingham Hearing Biomedical Research Unit, Otology and Hearing Group, Division of Clinical Neuroscience, School of Medicine, University of Nottingham, Nottingham, UK
[3]Nottingham University Hospitals NHS Trust, Nottingham, UK

*** Correspondence:** Dr Antje Heinrich, MRC Institute of Hearing Research, University Park, Nottingham, NG7 2RD, UK
antje.heinrich@ihr.ac.uk

1. Supplementary Data

To provide the reader with the full results, Supplementary Table 1 reports bivariate correlations between all speech and cognitive tests. As one of six speech tests demonstrated a significant correlation with age and four of six with hearing (BEA), Supplementary Table 2 provides the same speech-cognitive test correlations, but with age and hearing thresholds partialled out. Both tables demonstrate a consistent pattern with Phoneme Discrimination not correlating with any of the cognitive measures, word perception tests correlating with LNS mostly, and key word perception in a carrier sentence (MCRM) demonstrating the widest range of correlations with cognitive measures. Supplementary Table 3 provides Spearman correlation coefficients between self-reported ratings on residual disability in each of four listening situations and cognitive measures. Only two correlations, both with sustained attention, reach significance suggesting that the outcome of self-report measures is not intimately connected with cognitive abilities.

Supplementary Table 1. Simple Pearson product-moment correlations between each of six speech tests, age, hearing and cognitive tests. Phoneme in noise = Phoneme discrimination in noise; FAAF = Four Alternative Auditory Feature test; Words Q = single words presented in quiet; Words 0 = single words presented at 0 dB SNR; Words -4 = single words presented at -4 dB SNR; MCRM = Modified Coordinate Response Measure; BEA = better ear average$_{(0.5\text{-}4kHz)}$; TEA6/7 = Test of Everyday Attention subtests 6 & 7; TEA DTD = Test of Everyday Attention Dual Task Decrement; IMAP = IHR Multicentre study of Auditory Processing test; Digits = five digit encoding and recall; SICspan size = Size Comparison span, span size; SICspan intrusions = Size Comparison span, number of intrusions; LNS = Letter-Number Sequencing. *p < 0.05, **p < 0.01.

			Phonemes	Words				Sentences
			Discrimination in Noise	FAAF	Words			MCRM
					Q	0	-4	
	Age		.43*	-.14	-.31	-.17	.01	.31
	BEA		.08	-.64**	-.60**	-.60**	-.24	.41*
**Cognitive domain**	**Task**	**Sub-task**	Discrimination in Noise	FAAF	Words			MCRM
					Q	0	-4	
single attention	TEA6		-.05	-.06	-.25	-.22	.02	.46**
divided attention	TEA7		-.34	-.02	-.13	-.19	.21	.22
attention-related decrement	TEA DTD		-.15	-.13	.15	-.13	.05	-.12
Verbal WM under dual attention	Digit	Quiet	.08	.26	.07	.19	.39*	-.40*
		0 dB SNR	.08	-.06	-.15	.21	-.10	-.39*
		-4 dB SNR	.11	-.17	-.07	-.03	-.30	-.25
Sustained attention	IMAP	Visual uncued	.16	.22	-.12	-.05	-.01	.11
		Visual cued	.22	.08	-.20	-.16	-.09	.37*
		Audio uncued	.13	.08	-.33	-.05	-.02	.36*
		Audio cued	.12	-.09	-.36*	-.26	-.27	.46*
		Visual difference	.00	.28	.05	.11	.09	-.27
		Audio difference	.05	.23	-.07	.25	.31	-.02
Verbal WM and response control	SICspan	Size	-.06	.36*	.05	.18	.11	-.12
		Intrusions	-.24	-.17	-.24	-.12	.12	-.13
Verbal WM	LNS		.07	.62**	.67**	.56**	.54**	-.59**

Supplementary Table 2. Pearson product-moment correlations between the four speech tests and cognitive tests with hearing (BEA) partialled out. Acronyms as in Supplementary Table 1. *p < 0.05, **p < 0.01.

**Cognitive domain**	**Task**	**Sub-task**	Phoneme in Noise	FAAF	Words			MCRM
					Q	0	-4	
single attention	TEA6		-.04	-.17	-.42*	-.37	.01	.59**
divided attention	TEA7		-.34	-.08	-.26	-.30	.21	.28
attention-related decrement	TEA DTD		-.15	-.16	.07	-.15	.12	-.10
Verbal WM under dual attention and response control	Digit	Quiet	.08	.32	.09	.23	.40*	-.46*
		0dB	.07	-.05	-.14	.30	-.10	-.48*
		-4dB	.09	-.02	-.11	.16	-.27	-.40*
sustained attention	IMAP	Visual uncued	.17	.30	-.15	-.07	-.01	.13
		Visual cued	.21	.26	-.12	-.07	-.04	.34
		Audio uncued	.12	.23	-.31	-.04	.02	.34
		Audio cued	.10	.07	-.30	-.17	-.22	.43*
		Visual difference	.02	.18	-.11	-.03	.04	-.20
		Audio difference	.06	.27	-.12	.28	.31	-.00
Verbal WM and response control	SICspan	Size	-.06	.48**	.06	.22	.11	-.13
		Intrusions	-.25	-.13	-.21	-.06	.16	-.21
Verbal WM	LNS		.10	.57**	.65**	.49**	.50**	-.57**

Supplementary Table 3. Spearman correlation coefficients between self-rated residual disability, hearing sensitivity, and cognitive tests assessing various aspects of attention and WM. Acronyms as in Supplementary Table 1. *p < 0.05, **p < 0.01.

			GHABP			
			Q1	Q2	Q3	Q4
	Age		-.09	.32	.12	-.22
	BEA		.42*	-.05	.29	.28
Cognitive domain	Task	Sub-task	Q1	Q2	Q3	Q4
single attention	TEA6	TEA6	-.08	-.09	.12	.04
divided attention	TEA7	TEA7	.15	.02	.01	-.21
attention-related decrement	TEA DTD	DTD	.09	.04	-.17	-.27
Verbal WM under dual attention and response control	Digit	Quiet	.04	-.08	-.01	.02
		0 dB	.19	.14	.09	.08
		-4 dB	.32	-.09	.17	.11
sustained attention	IMAP	Visual uncued	-.07	.07	.06	-.09
		Visual cued	.30	.17	.17	.34
		Audio uncued	.10	.03	-.13	.32
		Audio cued	.36	.11	.22	.43*
		Visual difference	-.20	-.08	.05	-.28
		Audio difference	-.24	-.24	-.51**	.05
Verbal WM and response control	SICspan	Size	.13	.18	-.05	-.05
		Intrusions	-.05	-.30	.02	-.11
Verbal WM	LNS		-.16	-.07	-.32	-.24
